# HIV Trends in the United States: Diagnoses and Estimated Incidence

**DOI:** 10.2196/publichealth.7051

**Published:** 2017-02-03

**Authors:** H Irene Hall, Ruiguang Song, Tian Tang, Qian An, Joseph Prejean, Patricia Dietz, Angela L Hernandez, Timothy Green, Norma Harris, Eugene McCray, Jonathan Mermin

**Affiliations:** ^1^ Centers for Disease Control and Prevention Atlanta, GA United States; ^2^ ICF Atlanta, GA United States

**Keywords:** HIV infections, incidence, biomarkers, United States

## Abstract

**Background:**

The best indicator of the impact of human immunodeficiency virus (HIV) prevention programs is the incidence of infection; however, HIV is a chronic infection and HIV diagnoses may include infections that occurred years before diagnosis. Alternative methods to estimate incidence use diagnoses, stage of disease, and laboratory assays of infection recency. Using a consistent, accurate method would allow for timely interpretation of HIV trends.

**Objective:**

The objective of our study was to assess the recent progress toward reducing HIV infections in the United States overall and among selected population segments with available incidence estimation methods.

**Methods:**

Data on cases of HIV infection reported to national surveillance for 2008-2013 were used to compare trends in HIV diagnoses, unadjusted and adjusted for reporting delay, and model-based incidence for the US population aged ≥13 years. Incidence was estimated using a biomarker for recency of infection (stratified extrapolation approach) and 2 back-calculation models (CD4 and Bayesian hierarchical models). HIV testing trends were determined from behavioral surveys for persons aged ≥18 years. Analyses were stratified by sex, race or ethnicity (black, Hispanic or Latino, and white), and transmission category (men who have sex with men, MSM).

**Results:**

On average, HIV diagnoses decreased 4.0% per year from 48,309 in 2008 to 39,270 in 2013 (*P*<.001). Adjusting for reporting delays, diagnoses decreased 3.1% per year (*P*<.001). The CD4 model estimated an annual decrease in incidence of 4.6% (*P*<.001) and the Bayesian hierarchical model 2.6% (*P*<.001); the stratified extrapolation approach estimated a stable incidence. During these years, overall, the percentage of persons who ever had received an HIV test or had had a test within the past year remained stable; among MSM testing increased. For women, all 3 incidence models corroborated the decreasing trend in HIV diagnoses, and HIV diagnoses and 2 incidence models indicated decreases among blacks and whites. The CD4 and Bayesian hierarchical models, but not the stratified extrapolation approach, indicated decreases in incidence among MSM.

**Conclusions:**

HIV diagnoses and CD4 and Bayesian hierarchical model estimates indicated decreases in HIV incidence overall, among both sexes and all race or ethnicity groups. Further progress depends on effectively reducing HIV incidence among MSM, among whom the majority of new infections occur.

## Introduction

Annual estimates of the number of human immunodeficiency virus (HIV) infections in the United States peaked in the mid-1980s, decreased through the early 1990s, and remained relatively stable through 2010 [1,2]. Over time, with improved diagnosis and treatment, the number of people living with HIV has steadily increased and with that has come the potential for increased HIV transmission [3-5]. But knowledge of HIV infection is associated with decreased risk behavior, and the proportion of persons with HIV in the United States who know their status is at its highest ever [3,4]. Similarly, antiretroviral treatment (ART) for HIV substantially reduces the risk of viral transmission, and decreases in incidence have been observed in populations with higher uptake of ART [6-9]. Yet, it is unclear whether HIV prevention programs and ART use have resulted in decreasing HIV incidence in recent years in the United States.

A primary goal of the National HIV/AIDS Strategy for the United States is to reduce HIV incidence [10]. However, determining progress in reducing HIV incidence is challenging as direct measures are generally not available. Some recent reports suggest that HIV diagnoses decreased in the United States during the last decade [11-13]. However, HIV diagnoses trends are affected by testing rates, diagnoses delays, and incidence rates, and should therefore be interpreted in conjunction with data on HIV testing and available incidence estimates.

To assess recent progress toward reducing HIV infections in the United States overall and in selected population segments with available incidence estimation methods, we analyzed data reported to national surveillance programs at the Centers for Disease Control and Prevention (CDC). The data presented include case counts of HIV diagnoses as well as data from new and established models to estimate HIV incidence and testing data from behavioral surveys to aid interpretation of trends. In the United States, large disparities in HIV diagnoses exist among population segments; two-thirds of persons with HIV diagnosed each year are men who have sex with men (MSM), and blacks or African Americans are 8 times and Hispanics or Latinos 3 times as likely to be diagnosed with HIV as white Americans [14]. Therefore, assessment of progress toward reaching the goal of reducing HIV incidence should include trends for the United States overall and for disproportionately affected population segments.

## Methods

### HIV Diagnoses and Incidence Data Sources and Methods

Data from the National HIV Surveillance System reported to the CDC through December 2015 were used to determine trends in the annual number of HIV diagnoses in the United States [15]. Data on HIV diagnoses were also used to estimate the annual number of infections (incidence) with 3 models (Table 1) using (1) additional information on a biomarker that classifies infections as recent (or not) in the stratified extrapolation approach [1,2,16-18]; (2) HIV diagnoses and the severity of disease (whether infection is classified as stage-3 AIDS, within the same calendar year as HIV diagnosis) in the back-calculation approach (Bayesian hierarchical model) to estimate HIV prevalence and the percentage of persons living with undiagnosed HIV [4,19,20]; and (3) the first CD4 count after diagnosis in a newly developed approach to derive incidence, prevalence, and the percentage undiagnosed (CD4 model) [21-23]. The biomarker data required for the stratified extrapolation approach were collected by 18 states and 3 cities participating in the incidence surveillance component of the National HIV Surveillance System. Incidence was estimated for these combined areas and then extrapolated to the remaining areas of the United States [2]. For the Bayesian hierarchical model, input data were adjusted for underreporting of HIV diagnoses in the early years of the US epidemic period before HIV reporting was implemented by all jurisdictions, whereas information on AIDS diagnoses was available for all years [19]. For the CD4 model historical data were not required and data on diagnoses and CD4 test results were directly obtained from the surveillance data.

Data are presented for 2008 through 2013; starting in 2008, all states and the District of Columbia had implemented name-based HIV reporting and these cases were reported to the National HIV Surveillance System. Diagnoses and incidence estimates were adjusted for missing risk factor information and for determining whether reporting delays may affect the interpretation of trends, we conducted analyses unadjusted and adjusted for reporting delays [14].

Data on HIV diagnoses and for derived incidence include persons aged 13 years and older at the time of diagnosis or infection, respectively. Trends in these indicators were assessed overall and by sex and race or ethnicity (blacks or African Americans, (hereafter referred to as blacks); Hispanics or Latinos; and whites), and for MSM. HIV surveillance data can be considered to represent a census of HIV diagnoses for the United States and therefore no confidence intervals (CIs) are presented. For estimates of HIV incidence, 95% CIs were calculated. To determine whether there was a significant increasing or decreasing trend in the annual numbers of diagnoses or incidence, the estimated annual percent change (EAPC) in diagnoses and incidence and associated 95% CIs were calculated, and a change in trend was considered statistically significant if *P*<.05.

**Table 1:** Methods for estimating HIV incidence.

Name of the model	Stratified extrapolation approach [1,16,17]	Bayesian hierarchical model [19,20]	CD4 model [23]
Method	Biomarker-based sample survey	Bayesian-based back-calculation	CD4 based back-calculation
Data requirement	Data for single or multiple years, no limit on number of years	Data for entire epidemic period	Data for recent (8+) years
	All new diagnoses	All new diagnoses	All new diagnoses
	Incidence assay result on recency of infection	AIDS classification within year of diagnosis	First CD4 after diagnosis
	Testing and treatment history		
Strengths	Annual estimates	Annual estimates	Annual estimates
	More accurate for recent years		Data for entire epidemic period not required
Weaknesses	False recent rate of incidence assay used	HIV data in earlier years incomplete as jurisdictions implemented HIV reporting over time; hence relies on accuracy of data adjustment for incomplete reporting	Relies on accuracy of CD4 depletion model
	Relies on accuracy of testing and treatment information		

### HIV Testing Data Sources and Methods

Data on HIV testing among the US population are available from the National Health Interview Survey (NHIS) and the Behavioral Risk Factor Surveillance System (BRFSS), and these were used to determine trends in testing (a change in trend was considered statistically significant if *P*<.05). NHIS collects information on a broad range of health topics from a nationally representative sample of civilian, noninstitutionalized US households [24]. The annual NHIS response rate for the Sample Adult Survey ranged from 62.6% in 2008 to 60.8% in 2010 [24]. NHIS asks persons aged 18 years and above questions related to HIV testing (Have you ever been tested for HIV? In what month and year was your last test for HIV [the virus that causes AIDS]?). Differences observed in estimates of HIV testing based on NHIS 2010 and earlier and NHIS 2011 and later may be attributable to survey design changes and estimates for the percentage of persons ever tested are not comparable [25,26]. Therefore, the most recent years included in this analysis were 2008-2010. Only records for respondents aged 18-64 years were included, the age group for which CDC’s recommendations encourage HIV screening, and records had to have a “Yes” or “No” response to whether the respondent had ever been tested for HIV, excluding tests for blood donations.

BRFSS is a state-based, random-digit-dialed telephone (landline and mobile) survey of the civilian, noninstitutionalized adult US population that collects information on preventive health practices and risk behaviors. In 2011, BRFSS added mobile phone numbers to the sampling frame and implemented a new weighting methodology. Differences observed in estimates of HIV testing based on 2010 and earlier BRFSS and 2011 and later BRFSS may be attributable to these design changes and estimates of the percentage of persons ever tested during the 2 periods are not comparable [26]. The median weighted survey response rates for all states were 49.7% in 2011, 45.2% in 2012, and 45.9% in 2013 [27-29]. Ever tested for HIV and tested in the last year were based on respondents who reported having ever tested for HIV and whether the most recent HIV test was within a year of their BRFSS interview date. Analyses were weighted to account for the complex survey design, nonresponse, and sociodemographic factors to provide estimates of HIV testing that are representative of the civilian, noninstitutionalized population in the United States.

Data from National HIV Behavioral Surveillance (NHBS) for 2008, 2011, and 2014 were used to determine trends in HIV testing among MSM, ever and within the past 12 months. NHBS monitors HIV-associated behaviors in 20 cities with high AIDS burden [30]. A venue-based sampling method is used for the NHBS MSM cycles [31]. First, venues frequented by MSM (eg, bars, dance clubs, gyms, restaurants, parks, street locations, and social organizations) and days and times when men frequented those venues are identified. Second, venues and corresponding day-time periods were selected randomly for recruitment events. Third, men at recruitment events were systematically approached to screen for eligibility (aged ≥18 years, lived in a participating city, and able to complete the interview in English or Spanish). An additional eligibility criterion was applied in 2011 and 2014, by which only men who reported ever having sex with another man were eligible. Consent for participation in the survey was obtained and trained interviewers used handheld computers to administer a standardized anonymous questionnaire. All analyses were conducted using SAS version 9.3 statistical software (SAS Institute Inc), except for the Bayesian hierarchical model, which used R version 3.2.2 statistical software (The R Foundation for Statistical Computing).

## Results

### HIV Diagnoses, Incidence, and Testing Among the US Population

**Figure 1:**
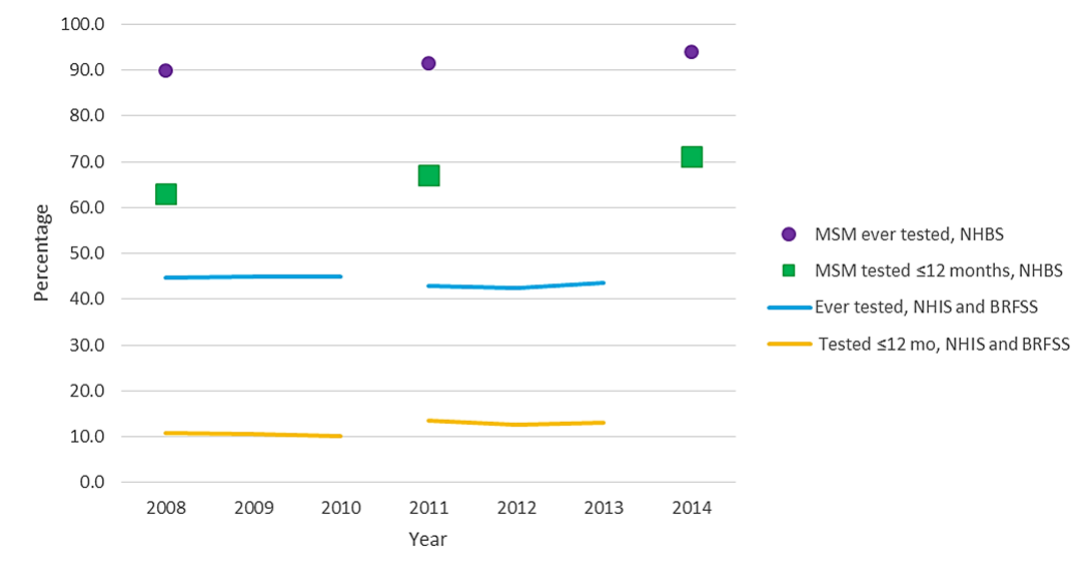
Percentage of persons reporting testing for HIV, United States, 2008-2014. HIV: human immunodeficiency virus; BRFSS: Behavioral Risk Factor Surveillance System; NHBS: National HIV Behavioral Surveillance; NHIS: National Health Interview Survey; MSM: men who have sex with men.

Annual diagnoses decreased from 48,309 in 2008 to 39,270 in 2013, an average rate of 4.0% per year, and diagnoses adjusted for reporting delays decreased 3.1% per year from 48,938 in 2008 to 41,625 in 2013 (Table 2). In 2013, depending on the model used, an estimated 34,400 (95% CI 27,700-39,000) to 36,300 (95% CI 34,000-38,500) persons were newly infected with HIV in the United States. The CD4 model estimated an annual decrease of 4.6% in new infections from 2008 to 2013. The Bayesian hierarchical model also estimated a decrease in infections (2.6% per year) whereas the stratified extrapolation approach estimated stable numbers of new infections. During these years, the number of persons who reported ever having received an HIV test or having had a test within the past 12 months remained stable (Figure 1).

**Table 2:** Number of diagnoses of HIV infection and HIV incidence, by selected characteristics, United States, 2008-2013.

Measure			Year						EAPC^a^	*P* value
			2008	2009	2010	2011	2012	2013		
**Total**										
	Diagnoses of HIV infection	No.	48,309	45,688	43,637	41,793	40,872	39,270	−4.0	<.001
		estimated No.^b^	48,938	46,428	44,564	43,043	42,686	41,625	−3.1	<.001
	Stratified extrapolation approach	No.	39,000	36,100	35,300	36,900	36,700	36,200	−0.7	.22
		95% CI	33,600	31,100	30,500	31,800	31,600	31,200		
			44,400	41,000	40,200	42,000	41,800	41,300		
	Bayesian hierarchical model	No.	39,700	37,100	36,200	35,600	35,200	34,400	−2.6	<.001
		95% CI	36,900	34,700	33,500	33,300	31,300	27,700		
			42,200	39,900	39,100	37,600	38,000	39,000		
	CD4 model	No.	46,000	43,900	41,600	40,000	38,300	36,300	−4.6	<.001
		95% CI	44,800	42,600	40,100	38,300	36,400	34,000		
			47,200	45,200	43,100	41,700	40,300	38,500		
**Black or African American**										
	Diagnoses of HIV infection	No.	22,702	21,325	20,214	19,108	18,348	17,517	−5.0	<.001
		estimated No.^b^	23,013	21,695	20,669	19,722	19,234	18,666	−4.1	<.001
	Stratified extrapolation approach	No.	17,600	15,400	14,800	16,200	15,200	15,600	−1.5	.09
		95% CI	15,000	13,200	12,600	13,800	12,900	13,300		
			20,200	17,600	17,000	18,500	17,400	17,900		
	Bayesian hierarchical model	No.	18,700	16,700	15,900	16,100	16,100	15,900	−3.1	<.001
		95% CI	16,400	14,700	14,200	14,200	14,000	11,500		
			21,300	18,700	17,200	17,800	19,800	21,400		
	CD4 model	No.	21,600	20,700	19,300	18,300	17,000	16,100	−5.7	<.001
		95% CI	20,700	19,700	18,200	17,100	15,700	14,500		
			22,400	21,600	20,300	19,500	18,300	17,600		
**Hispanic or Latino**										
	Diagnoses of HIV infection	No.	9801	9466	9158	8998	8997	8788	−2.0	<.001
		estimated No.^b^	9928	9615	9351	9263	9389	9299	−1.2	<.001
	Stratified extrapolation approach	No.	7900	7600	7600	8100	8000	8100	1.0	.40
		95% CI	6600	6300	6400	6800	6700	6800		
			9200	8800	8800	9300	9200	9500		
	Bayesian hierarchical model	No.	8100	8000	8100	8300	8200	8100	0.4	.22
		95% CI	7200	6500	7000	7100	6100	5300		
			8900	8900	9100	9800	10,200	10,500		
	CD4 model	No.	9500	9200	8800	8700	8700	8600	−2.2	.05
		95% CI	8900	8600	8100	7800	7700	7400		
			10,000	9900	9500	9500	9700	9700		
**White**										
	Diagnoses of HIV infection	No.	13,109	12,327	11,768	11,262	11,142	10,708	−3.8	<.001
		estimated No.^b^	13,264	12,506	11,993	11,559	11,574	11,275	−3.1	<.001
	Stratified extrapolation approach	No.	11,100	10,900	10,800	10,400	11,100	10,600	−0.6	.63
		95% CI	9300	9100	9100	8700	9400	8900		
			12,900	12,600	12,500	12,100	12,900	12,300		
	Bayesian hierarchical model	No.	11,100	10,200	10,000	10,000	10,100	9800	−2.1	<.001
		95% CI	10,000	8300	9000	8500	7800	6500		
			12,300	11,200	10,900	12,000	12,300	12,600		
	CD4 model	No.	12,400	11,500	11,100	10,700	10,400	9500	−4.7	<.001
		95% CI	11,900	10,900	10,400	9900	9500	8400		
			13,000	12,100	11,800	11,400	11,300	10,500		

^a^EAPC: estimated annual percent change.

^b^Numbers are adjusted for reporting delays.

### HIV Diagnoses, Incidence, and Testing Among Population Segments

Among blacks, the number of HIV diagnoses decreased 5.0% per year from 2008 to 2013 (4.1% for diagnoses adjusted for reporting delays; Table 2). Among Hispanics or Latinos and whites, diagnoses decreased 2.0% (adjusted, 1.2%) and 3.8% (adjusted, 3.1%) per year, respectively. The CD4 model indicated decreases in incidence among blacks, Hispanics or Latinos, and whites, whereas the Bayesian hierarchical model indicated decreases among blacks and whites and the stratified extrapolation approach indicated that HIV incidence remained stable among all race or ethnicity groups.

**Figure 2:**
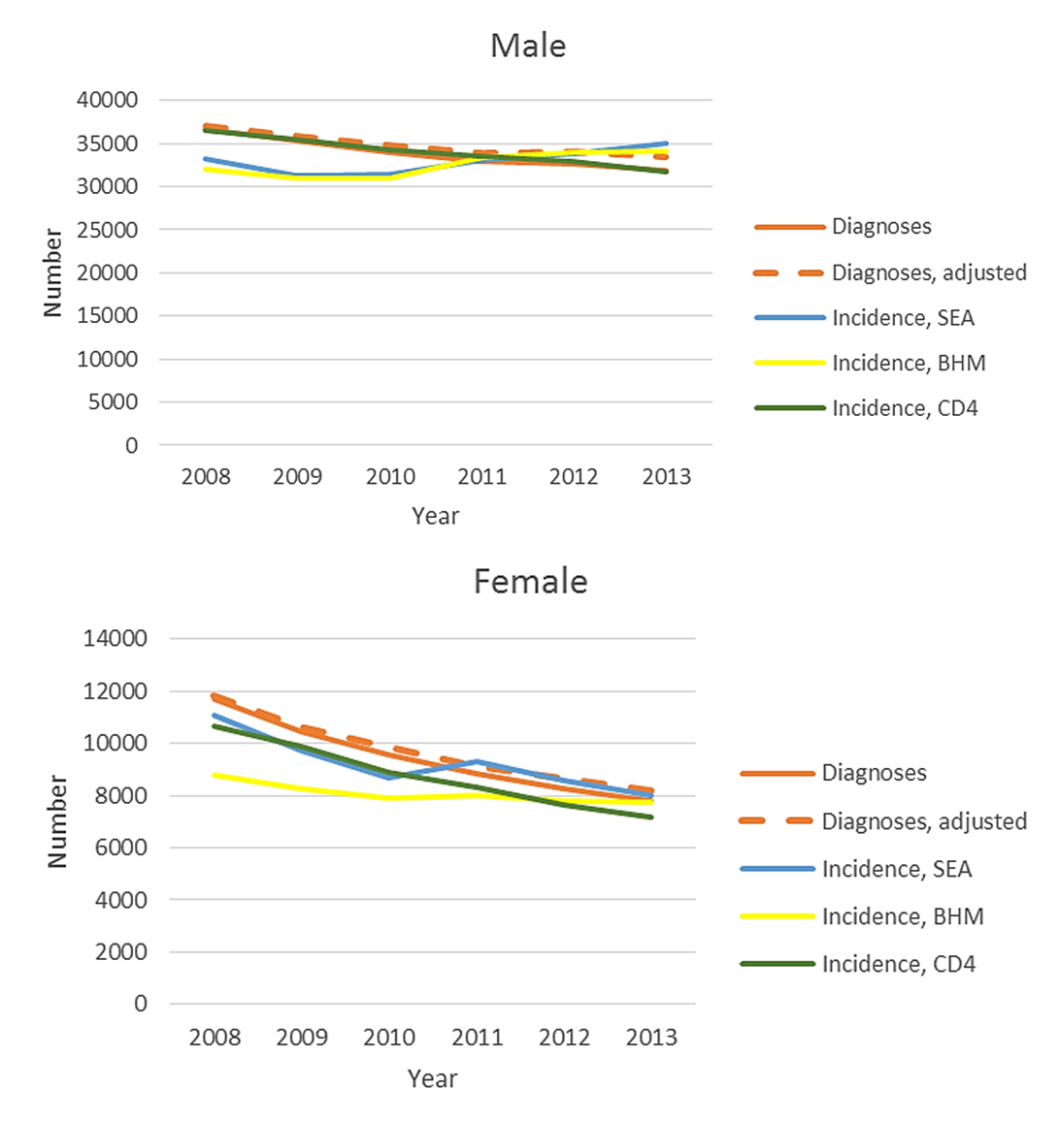
Number of diagnoses of HIV infection and estimated HIV infections, by sex, United States, 2008-2013. HIV: human immunodeficiency virus; BHM: Bayesian hierarchical model; CD4: CD4 model; SEA: stratified extrapolation approach.

Among males, the number of diagnoses decreased 2.8% per year from 2008 (36,614 diagnoses) to 2013 (31,578 diagnoses; decrease in adjusted diagnoses, 2.0%; Figure 2). Trends in estimated new HIV infections among males were inconsistent between the models. Incidence decreased by 3.5% (95% CI −4.6% to −2.4%) per year based on the CD4 model (2008: 35,600 infections, 95% CI 34,500-36,600; 2013: 29,600 infections, 95% CI 27,500-31,700), and by 1.5% (95% CI −1.9% to −1.2%) based on the Bayesian hierarchical model (2008: 31,500 infections, 95% CI 28,200-33,900; 2013: 28,900 infections, 95% CI 22,100-33,400). Based on the stratified extrapolation approach, HIV incidence remained stable among males (EAPC 0.9%, 95% CI −0.5% to 2.2%) from 2008 (29,400 infections, 95% CI 25,300-33,500) to 2013 (29,800 infections, 95% CI 25,700-34,000). The number of HIV diagnoses and infections among females decreased by about 30% between 2008 and 2013 using any measure, with average annual decreases in incidence from 4.2% to 8.7%.

**Figure 3:**
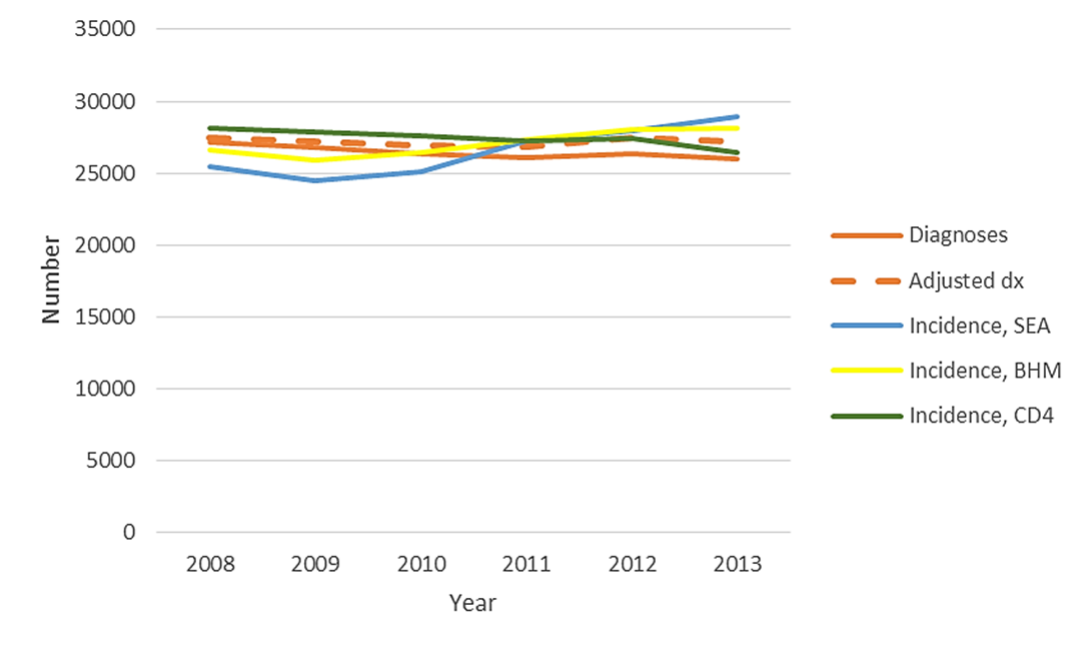
Number of diagnoses of HIV infection and estimated HIV infections among MSM, United States, 2008-2013. HIV: human immunodeficiency virus; BHM: Bayesian hierarchical model; CD4: CD4 model; SEA: stratified extrapolation approach; MSM: men who have sex with men.

Among men with infection attributed to male-to-male sexual contact, who accounted for 81.3% of males with HIV diagnosed in 2013, the number of HIV diagnoses decreased by 1.0% per year from 2008 (27,119 diagnoses) to 2013 (25,670 diagnoses), with no significant decrease observed in the diagnoses adjusted for reporting delays (Figure 3). During that time, the percentage of MSM who reported testing for HIV within the past 12 months increased from 63.0% in 2008 to 71.1% in 2014 (*P*<.001; Figure 1). More than 90% of MSM reported ever testing for HIV in recent years. The number of new infections among MSM increased by 2.5% per year (95% CI 1.0%-4.0%) based on the stratified extrapolation approach (2008: 22,600 infections, 95% CI 19,400-25,800; 2013: 24,700 infections, 95% CI 21,200-28,200), but the CD4 model (EAPC −1.8%, 95% CI −3.0% to −0.5%; 2008: 27,400, 95% CI 26,500-28,200; 2013: 24,600, 95% CI 22,700-26,500) and Bayesian hierarchical model (EAPC −2.5%, 95% CI −2.9% to −2.1%; 2008: 25,700 infections, 95% CI 24,000-27,700; 2013: 22,800 infections, 95% CI 19,000-26,800) both indicated a decrease in HIV incidence (Figure 3).

### HIV Incidence Adjusted for Reporting Delay

When analyses were repeated with data adjusted for delays in reporting of HIV diagnoses to the National HIV Surveillance System, the findings varied across models and population segments. For all 3 models, incidence estimates based on data adjusted for reporting delays did not change the interpretation of trends for blacks, whites, and females (data not shown). The Bayesian hierarchical model indicated a small increase in incidence overall (EAPC 0.8%, 95% CI 0.5%-1.1%) and among Hispanics or Latinos (EAPC 3.9%, 95% CI 3.2%-4.5%). For men with infection attributed to male-to-male sexual contact, estimates based on data adjusted for reporting delays from the CD4 (EAPC −1.0%, 95% CI −2.2% to 0.3%) and the Bayesian hierarchical models (EAPC 0.66%, 95% CI 0.31%-1.01%) no longer indicated a decrease in incidence.

## Discussion

### Principal Findings

The study findings are that diagnoses of HIV infection and incidence estimates from 2 models indicate a reduction in HIV incidence from 2008 through 2013 overall and in subpopulations, including women, men, and MSM. Compared with earlier estimates of the number of new infections in the United States [1,2,17], HIV incidence decreased from about 50,000 infections in the 1990s through the mid-2000s to around 36,000 infections in 2013. For MSM, previously published estimates [1,2] indicate an increase in incidence from about 20,000 infections in the early 1990s to about 30,000 infections in the mid-2000s, with relatively stable incidence thereafter and, based on our analyses, about 25,000 infections in 2013. Our results from the stratified extrapolation approach for 2008-2010 are lower than the previously reported incidence estimates for these years (47,500, 45,000, and 47,000, respectively) based on the same model [2]. There is new evidence that the mean recency period (an estimate of the time between seroconversion and the time the biomarker reaches a value defined as distinguishing recent vs long-standing infection) of the BED assay is longer than previously estimated [18]. Use of a shorter recency period in the past resulted in an overestimation of incidence and therefore, a revision to modeling with the stratified extrapolation approach was required. We revised the method using the newly estimated recency period of 198 days for the BED assay (compared with 162 days used in the past) [18].

Our analyses indicated substantial reductions in HIV incidence in the United States, including among blacks and Hispanics or Latinos, who are disproportionately affected by HIV. The results also suggest modest reductions among MSM, a population with a considerably higher HIV prevalence than heterosexuals, indicating the need for greater reach of HIV prevention services to make substantial reductions in incidence. HIV testing appears to be increasing among MSM, potentially due to large-scale national efforts, with a high and increasing proportion ever tested for HIV and more MSM tested within the past 12 months. This may be reflected in previously reported increases in HIV diagnoses among young MSM who are most likely to have undiagnosed HIV, and the overall increase in awareness of HIV infection among MSM [4,11]. Annual testing is recommended for sexually active MSM and more frequent testing may be indicated for those at highest risk for HIV infection to detect HIV infection early, which allows risk counseling and initiation of treatment and is cost effective [32-34]. Additional assessments are needed to determine whether testing is not reaching certain subpopulations of MSM at high risk for HIV infection.

More work needs to be done to alleviate the possible reasons that HIV transmission continues at high rates among MSM, including a proportion of MSM with viral suppression well short of national goals [4,10], increases in risk behavior [35], and lack of substantial uptake of pre-exposure prophylaxis (PrEP) to date [36]. The overall high proportion of undiagnosed HIV (51% in 2013) among young persons may contribute to higher HIV transmission [4]. In addition, the proportion with a suppressed viral load is lower among younger compared with older MSM [37]. With MSM accounting for the majority of transmissions of HIV in the United States [14,38], it is crucial that prevention efforts reach all MSM.

Blacks and Hispanics or Latinos remain disproportionately affected by HIV compared with whites. In 2013, about 44% of persons who were infected with HIV were black and about 24% Hispanic or Latino, compared with them comprising 12% and 17% of the US population, respectively. The decreasing trends in diagnoses and incidence among women are encouraging and, as previously reported, are mirrored by decreasing diagnoses among black, Hispanic or Latino, and white women [39]. Data on HIV testing in the United States for women overall do not indicate that decreases in diagnoses among women would be due to decreases in testing [40]. However, some data indicate that testing among young women, including black and white young women, has decreased from 2011 to 2013 [41], whereas data for women at increased risk for HIV infection from NHBS indicate an increase in testing [42,43]. To achieve the goal of the National HIV/AIDS Strategy of reducing disparities in HIV, there is a need to strengthen treatment for persons living with HIV to improve their health and prevent transmission, as well as primary prevention efforts [10]. Lower percentages of blacks and Hispanics or Latinos living with HIV have their infection diagnosed or are promptly linked to care after diagnosis [4]. Disparities by race or ethnicity also exist in receipt of treatment and viral suppression overall as well as among women and MSM [4,37,39].

### Limitations

There are some limitations to each of the measures available to estimate trends in HIV incidence. Diagnoses represent a census of events for the United States. However, trends in diagnoses depend on testing rates and are subject to diagnosis delays, with an estimated median delay between HIV infection and HIV diagnosis of 3.6 years (mean 5.6 years) for 2011 [21]. Back-calculation models to estimate incidence rely on valid input data on diagnoses and the time from infection to late stage disease (Table 1). An advantage to the CD4 model is that historical data are not required. The Bayesian hierarchical model, on the other hand, requires input data for the entire epidemic period and hence additional uncertainty is introduced because of the need to estimate HIV cases for the early years when HIV testing was not available and when few jurisdictions had HIV reporting even after HIV testing became available. Another advantage of the CD4 model is that HIV surveillance requires the reporting of the first CD4 count after HIV diagnosis in all jurisdictions, and CD4 reporting completeness is expected to increase as laboratory reporting improves [4]. Collection of biomarker data for the stratified extrapolation approach is limited to the 18 states and 3 cities participating in incidence surveillance, requiring extrapolation to the remaining areas of the United States [2]. The stratified extrapolation approach is also subject to incidence assay and testing history inaccuracies [44]. In addition, stratified extrapolation approach estimates rely on a correctly calculated mean recency period for the incidence assay used. We applied the updated mean recency period of 198 days for the BED assay (compared with 162 days used in the past) [18], which resulted in lower incidence estimates compared with earlier estimates. These limitations may also explain why the incidence estimates from the stratified extrapolation approach were at times inconsistent with the other 2 methods. Back-calculation models have greater uncertainty in later years reflected in wider CIs, and hence more uncertainty in determining trends. Estimating incidence by age with back-calculation models is more complex as age at HIV infection must also be estimated but could be included in future work. Reporting of HIV diagnoses is subject to reporting delays and duplicate reporting of cases, which primarily affect the reporting of data for the most recent years. Therefore, adjustment for reporting delays may overestimate diagnoses when duplicate cases have not been removed from the data. Finally, long-term trend data on testing rates to compare with diagnosis trends are not available for the general population or the entire population of MSM. The NHBS System relies on venue-based, time-space sampling of MSM in 20 large urban areas and therefore may not be representative of the entire MSM population. Testing data are also subject to accuracy of recall and possibly response influenced by social desirability.

### Conclusions

In summary, incidence models estimated that about 36,000 people were infected with HIV in the United States in 2013. From 2008 to 2013, HIV diagnoses decreased overall, among both sexes and all race or ethnicity groups, and similar to earlier estimates of HIV incidence [45,46], the CD4 and Bayesian hierarchical models indicated decreases in incidence. The overall decrease in incidence reflects a substantial decrease among women, heterosexual men, and as previously reported, among persons who inject drugs [1,2]. However, further progress is dependent on effectively reducing HIV incidence among MSM, among whom the majority of new infections occur. To do so, the nation will need to accelerate access to testing, antiretroviral therapy, and prevention advances, including PrEP, to reduce HIV infections by the targeted 25% of the National HIV/AIDS Strategy [10,47,48].

## Abbreviations

ART: antiretroviral treatment
BRFSS: Behavioral Risk Factor Surveillance System
EAPC: estimated annual percent change
HIV: human immunodeficiency virus
MSM: men who have sex with men
NHIS: National Health Interview Survey
NHBS: National HIV Behavioral Surveillance

## References

[1] Hall HI, Song R, Rhodes P, Prejean J, An Q, Lee LM, Karon J, Brookmeyer R, Kaplan EH, McKenna MT, Janssen RS, HIV Incidence Surveillance Group (2008). Estimation of HIV incidence in the United States. J Am Med Assoc.

[2] Centers for Disease Control and Prevention (2012). CDC.

[3] Chen M, Rhodes PH, Hall IH, Kilmarx PH, Branson BM, Valleroy LA (2012). Prevalence of undiagnosed HIV infection among persons aged ≥13 years--National HIV Surveillance System, United States, 2005-2008. MMWR Morb Mortal Wkly Rep.

[4] Centers for Disease Control and Prevention (2016). CDC.

[5] (2015). Aidsinfo.nih.

[6] Cohen MS, Chen YQ, McCauley M, Gamble T, Hosseinipour MC, Kumarasamy N, Hakim JG, Kumwenda J, Grinsztejn B, Pilotto JH, Godbole SV, Mehendale S, Chariyalertsak S, Santos BR, Mayer KH, Hoffman IF, Eshleman SH, Piwowar-Manning E, Wang L, Makhema J, Mills LA, de Bruyn G, Sanne I, Eron J, Gallant J, Havlir D, Swindells S, Ribaudo H, Elharrar V, Burns D, Taha TE, Nielsen-Saines K, Celentano D, Essex M, Fleming TR, HPTN 052 Study Team (2011). Prevention of HIV-1 infection with early antiretroviral therapy. N Engl J Med.

[7] Attia S, Egger M, Müller M, Zwahlen M, Low N (2009). Sexual transmission of HIV according to viral load and antiretroviral therapy: systematic review and meta-analysis. AIDS.

[8] Montaner JSG, Lima VD, Barrios R, Yip B, Wood E, Kerr T, Shannon K, Harrigan PR, Hogg RS, Daly P, Kendall P (2010). Association of highly active antiretroviral therapy coverage, population viral load, and yearly new HIV diagnoses in British Columbia, Canada: a population-based study. Lancet.

[9] Tanser F, Bärnighausen T, Grapsa E, Zaidi J, Newell M (2013). High coverage of ART associated with decline in risk of HIV acquisition in rural KwaZulu-Natal, South Africa. Science.

[10] The White House Office of National AIDS Policy (2015). AIDS.

[11] Johnson AS, Hall HI, Hu X, Lansky A, Holtgrave DR, Mermin J (2014). Trends in diagnoses of HIV infection in the United States, 2002-2011. J Am Med Assoc.

[12] Torian LV, Forgione LA (2015). Young MSM at the leading edge of HIV in New York City: back to the future?. J Acquir Immune Defic Syndr.

[13] Frieden TR, Foti KE, Mermin J (2015). Applying public health principles to the HIV epidemic--how are we doing?. N Engl J Med.

[14] Centers for Disease Control and Prevention (2016). CDC.

[15] Cohen SM, Gray KM, Ocfemia MCB, Johnson AS, Hall HI (2014). The status of the National HIV Surveillance System, United States, 2013. Public Health Rep.

[16] Karon JM, Song R, Brookmeyer R, Kaplan EH, Hall HI (2008). Estimating HIV incidence in the United States from HIV/AIDS surveillance data and biomarker HIV test results. Stat Med.

[17] Prejean J, Song R, Hernandez A, Ziebell R, Green T, Walker F, Lin LS, An Q, Mermin J, Lansky A, Hall HI, HIV Incidence Surveillance Group (2011). Estimated HIV incidence in the United States, 2006-2009. PLoS One.

[18] Hanson DL, Song R, Masciotra S, Hernandez A, Dobbs TL, Parekh BS, Owen SM, Green TA (2016). Mean recency period for estimation of HIV-1 incidence with the BED-Capture EIA and Bio-Rad Avidity in persons diagnosed in the United States with subtype B. PLoS One.

[19] Hall HI, An Q, Tang T, Song R, Chen M, Green T, Kang J (2015). Prevalence of Diagnosed and Undiagnosed HIV Infection--United States, 2008-2012. MMWR Morb Mortal Wkly Rep.

[20] An Q, Kang J, Song R, Hall HI (2016). A Bayesian hierarchical model with novel prior specifications for estimating HIV testing rates. Stat Med.

[21] Hall HI, Song R, Szwarcwald CL, Green T (2015). Brief report: Time from infection with the human immunodeficiency virus to diagnosis, United States. J Acquir Immune Defic Syndr.

[22] Song R, Landmann SC, Green TA, Hall HI (2014). Estimating HIV incidence, prevalence, and proportion undiagnosed based on CD4 data.

[23] Song R, Hall HI, Green TA, Szwarcwald CL, Pantazis N (2017). Using CD4 data to estimate HIV incidence, prevalence, and percent of undiagnosed infections in the United States. J Acquir Immune Defic Syndr.

[24] National Center for Health Statistics (2014). CDC.

[25] National Center for Health Statistics (2014). CDC.

[26] Van Handel M, Branson BM (2014). The consequences of methodology changes to national surveys on monitoring HIV testing trends in the United States.

[27] National Center for Chronic Disease Prevention and Health Promotion (2013). CDC.

[28] National Center for Chronic Disease Prevention and Health Promotion (2013). CDC.

[29] National Center for Chronic Disease Prevention and Health Promotion (2015). CDC.

[30] Gallagher KM, Sullivan PS, Lansky A, Onorato IM (2007). Behavioral surveillance among people at risk for HIV infection in the U.S.: the National HIV Behavioral Surveillance System. Public Health Rep.

[31] MacKellar DA, Gallagher KM, Finlayson T, Sanchez T, Lansky A, Sullivan PS (2007). Surveillance of HIV risk and prevention behaviors of men who have sex with men--a national application of venue-based, time-space sampling. Public Health Rep.

[32] Branson BM, Handsfield HH, Lampe MA, Janssen RS, Taylor AW, Lyss SB, Clark JE, Centers for Disease Control and Prevention (CDC) (2006). Revised recommendations for HIV testing of adults, adolescents, and pregnant women in health-care settings. MMWR Recomm Rep.

[33] Oster Am, Miles IW, Le BC, DiNenno EA, Wiegand RE, Heffelfinger JD, Wolitski R (2011). HIV testing among men who have sex with men--21 cities, United States, 2008. MMWR Morb Mortal Wkly Rep.

[34] Hutchinson AB, Farnham PG, Sansom SL, Yaylali E, Mermin JH (2016). Cost-effectiveness of frequent HIV testing of high-risk populations in the United States. J Acquir Immune Defic Syndr.

[35] Paz-Bailey G, Hall HI, Wolitski RJ, Prejean J, Van Handel MM, Le B, LaFlam M, Koenig LJ, Mendoza MCB, Rose C, Valleroy LA (2013). HIV testing and risk behaviors among gay, bisexual, and other men who have sex with men - United States. MMWR Morb Mortal Wkly Rep.

[36] Smith DK, Van Handel MM, Wolitski RJ, Stryker JE, Hall HI, Prejean J, Koenig LJ, Valleroy LA (2015). Vital signs: estimated percentages and numbers of adults with indications for Preexposure Prophylaxis to prevent HIV acquisition--United States, 2015. Morb Mortal Wkly Rep.

[37] Singh S, Bradley H, Hu X, Skarbinski J, Hall HI, Lansky A (2014). Men living with diagnosed HIV who have sex with men: progress along the continuum of HIV care--United States, 2010. MMWR Morb Mortal Wkly Rep.

[38] Oster AM, Wertheim JO, Hernandez AL, Ocfemia MCB, Saduvala N, Hall HI (2015). Using molecular HIV surveillance data to understand transmission between subpopulations in the United States. J Acquir Immune Defic Syndr.

[39] Nwangwu-Ike N, Hernandez AL, An Q, Huang T, Hall HI (2015). The epidemiology of human immunodeficiency virus infection and care among adult and adolescent females in the United States, 2008-2012. Womens Health Issues.

[40] National Center for HIV/AIDS, Viral Hepatitis, STD, and TB Prevention (2013). CDC.

[41] Van Handel M, Kann L, Olsen EO, Dietz P (2016). HIV testing among US high school students and young adults. Pediatrics.

[42] Centers for Disease Controland Prevention (2016). CDC.

[43] Sionean C, Le BC, Hageman K, Oster AM, Wejnert C, Hess KL, Paz-Bailey G, Centers for Disease Control and Prevention (CDC) (2014). HIV Risk, prevention, and testing behaviors among heterosexuals at increased risk for HIV infection--National HIV Behavioral Surveillance System, 21 U.S. cities, 2010. MMWR Surveill Summ.

[44] (2006). UNAIDS Reference Group on estimates, modelling and projections--statement on the use of the BED assay for the estimation of HIV-1 incidence for surveillance or epidemic monitoring. Wkly Epidemiol Rec.

[45] Bonacci RA, Holtgrave DR (2016). Evaluating the impact of the US National HIV/AIDS strategy, 2010-2015. AIDS Behav.

[46] Xia Q, Teixeira-Pinto A, Forgione LA, Wiewel EW, Braunstein SL, Torian LV (2017). Estimated HIV incidence in the United States, 2003-2010. J Acquir Immune Defic Syndr.

[47] National Center for HIV/AIDS, Viral Hepatitis, STD, and TB Prevention (2016). CDC.

[48] Crowley JS, Feirman S, Collins C, Holtgrave DR (2015). Generating hypotheses to explain declining HIV infection in four U.S. jurisdictions. AIDS Educ Prev.

